# TIRAP, TRAM, and Toll-Like Receptors: The Untold Story

**DOI:** 10.1155/2023/2899271

**Published:** 2023-03-07

**Authors:** Valérie Lannoy, Anthony Côté-Biron, Claude Asselin, Nathalie Rivard

**Affiliations:** Department of Immunology and Cell Biology, Cancer Research Institute, Faculty of Medicine and Health Sciences, Université de Sherbrooke, Sherbrooke, Québec, Canada

## Abstract

Toll-like receptors (TLRs) are the most studied receptors among the pattern recognition receptors (PRRs). They act as microbial sensors, playing major roles in the regulation of the innate immune system. TLRs mediate their cellular functions through the activation of MyD88-dependent or MyD88-independent signaling pathways. Myd88, or myeloid differentiation primary response 88, is a cytosolic adaptor protein essential for the induction of proinflammatory cytokines by all TLRs except TLR3. While the crucial role of Myd88 is well described, the contribution of other adaptors in mediating TLR signaling and function has been underestimated. In this review, we highlight important results demonstrating that TIRAP and TRAM adaptors are also required for full signaling activity and responses induced by most TLRs.

## 1. Introduction

The history of Toll-like receptors (TLRs) over the last 30 years begins with the discovery of the *Toll* gene responsible for *Drosophila* dorsoventral patterning during development. This was followed by the discovery, in 1996, that *Drosophila* Toll is involved in antifungal responses [1]. Since then, TLRs have been identified in invertebrates and vertebrates, including mammals, and their role in innate immunity has been extensively studied. The first mammalian homolog of *Drosophila* Toll was identified in 1997 as hToll, now termed TLR4 [2]. Today, the TLR family includes ten members in human (TLR1-TLR10) and twelve in mouse (TLR1-TLR9 and TLR11-TLR13) [3].

TLRs belong to the innate immunity receptor superfamily pattern recognition receptors (PRRs) [4]. TLRs consist of a cytoplasmic Toll-interleukin-1 receptor (TIR) domain conserved between TLR and the interleukin-1 receptor (IL-1R) families, as well as extracellular leucine-rich repeats (LRRs) [5]. Ubiquitously expressed [6], TLRs detect specific microbe-, pathogen-, and damage-associated molecular patterns, respectively, named MAMPs, PAMPs, and DAMPs, through LRR motif binding [7]. TLR-dependent recognition of microbial components triggers innate immune activation by regulating proinflammatory gene expression, among others. Individual TLRs differentially distributed within the cell interact with specific microbial-derived ligands. For example, TLR1, TLR2, TLR4, TLR5 and TLR6 are expressed on the cell surface and recognize conserved motifs on extracellular microorganisms like bacteria, fungi or protozoa [8]. In contrast, TLR3, TLR7, TLR8, and TLR9 are mostly expressed in the endo-lysosomal compartments [9–11]. During viral infection, receptor-mediated virus entry is usually directed to the cytoplasm, but occasionally, the virus enters the endosomal compartment. This may result in viral particle degradation, causing endosomal TLR ligand exposure to double- and single-stranded ribonucleic acids (dsRNAs and ssRNAs), which are TLR3 and TLR7/8 ligands, respectively [12].

**Figure 1:**
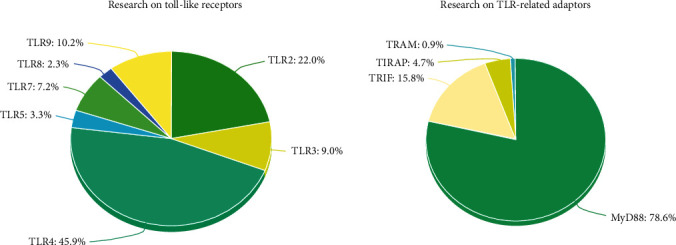
TLR4 and MyD88 hegemony in TLR research. The number of publications regarding each TLR and TLR-related adaptor referenced in PubMed® was calculated on 27^th^ October 2022. 28,034 publications regarding TLR4 were found; 13,431 on TLR2; 6,237 on TLR9; 5,516 on TLR3; 4,426 on TLR7; 2,011 on TLR5; 1,435 on TLR8; 10,148 on MyD88; 2,037 on TRIF; 601 on TIRAP; and 118 on TRAM.

Microbial motif recognition promotes TLR dimerization. TLR2 forms a heterophilic dimer with TLR1 or TLR6, while TLRs may form homodimers in other cases [13]. TLR dimerization activates signaling pathways that originate from the conserved intracellular TIR domain. Downstream of TIR, the TIR domain-containing adaptor myeloid differentiation primary response 88 (MyD88), is essential for the induction of proinflammatory cytokines, such as tumor necrosis factor-*α* (TNF-*α*) and interleukin-12 (IL-12) by all TLRs, except TLR3 [14]. Notably, TLR signaling operates through MyD88-dependent or MyD88-independent pathways. While a major role for Myd88 in mediating TLR signaling and function has been well described [15], the contribution of other signaling adaptors has been underestimated (Figure 1).

In this review, we highlight important results suggesting that TIRAP and TRAM adaptors are also required for full signaling activity and responses induced by most TLRs.

## 2. TLR Adaptors: An Overview

### 2.1. MyD88

MyD88, the universal adaptor protein for all TLRs except TLR3, triggers the activation of the proinflammatory nuclear factor-*κ*B (NF-*κ*B) pathway. Inflammatory cytokines are not induced in *MyD88*-deficient mice in response to stimulation by all TLRs but TLR3 [16]. MyD88 contains a N-terminal death domain (DD) and a C-terminal TIR domain [17], which associates with the TLR intracellular TIR domain after ligand stimulation. MyD88 recruits IL-1 receptor-associated kinase 4 (IRAK4) through DD-DD interactions and facilitates IRAK4-mediated phosphorylation of IRAK1 [18] for TNF receptor-associated factor 6 (TRAF6) engagement. Both IRAK1 and TRAF6 are polyubiquitinated in response to TLR agonists, then activating mitogen-activated protein kinases (MAPK) and NF-*κ*B signaling pathways [19].

### 2.2. TIR Domain-Containing Adaptor Protein (TIRAP)

**Figure 2:**
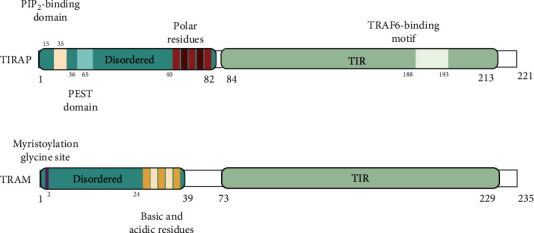
Structural view of human TIRAP and TRAM adaptor proteins. TIRAP contains a N-terminal PIP_2_-binding motif and a PEST domain, allowing polyubiquitination for rapid proteasomal degradation through suppressor of cytokine signaling 1 (SOCS1) binding [20]. TIRAP contains a C-terminal TIR domain. Its TRAF-6 binding motif permits direct association with TRAF6 for activation [21]. TRAM contains a N-terminal bipartite sorting signal that comprises its myristylation glycine site and controls its trafficking between the plasma membrane and the endosomes [22]. Similar to TIRAP, TRAM contains a C-terminal TIR domain.

**Figure 3:**
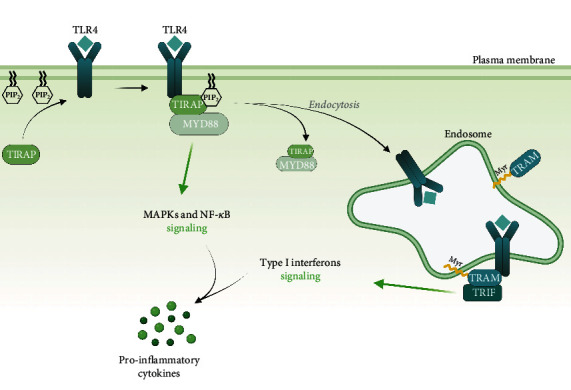
Schematic representation of the adaptors that bind the TIR domain of TLRs. TIRAP is preferentially localized at the cytoplasmic membrane through a PIP_2_-binding domain and recruits MyD88. Myristoylated TRAM localizes at the endosomes and triggers IFN I production via TRIF. Abbreviations: MAPKs: mitogen-activated protein kinases; MyD88: myeloid differentiation primary response 88; NF-*κ*B: nuclear factor-*κ*B; PIP_2_: phosphatidylinositol bisphosphate; TIRAP: Toll/interleukin-1 receptor domain-containing adaptor protein; TLR: Toll-like receptor; TRAM: TRIF-related adaptor molecule; TRIF: TIR domain-containing adaptor-inducing interferon-*β*.

The search for Myd88 structurally related proteins identified TIRAP [23]. Similar to *MyD88*-deficient macrophages, *Tirap*-deficient macrophages are impaired for cytokine production, following TLR2 and TLR4 stimulation [24]. The activation of MyD88-dependent pathways requires the TIRAP adaptor to bridge MyD88 to TLR4 [25]. While TIRAP bears a C-terminal TIR domain for TLR interaction (Figure 2), TIRAP lacks a motif to associate with downstream signaling effectors, as opposed to MyD88 which possesses a DD domain [26]. Importantly, TIRAP carries a N-terminal phosphatidylinositol-4,5 bisphosphate (PIP_2_) binding motif enabling its recruitment to the plasma membrane [27]. Indeed, TLR4 initially recruits TIRAP at the plasma membrane, then MyD88, triggering NF-*κ*B and MAPK pathways. Subsequently, TLR4 endocytosis and depletion of PIP_2_ from the plasma membrane release TLR4 from the TIRAP-MyD88 complex [28, 29]. This allows TLR4 to associate with TRIF-related adaptor molecule (TRAM) and then TIR-domain-containing adaptor-inducing interferon-*β* (TRIF) (Figure 3) for endosomal signaling.

### 2.3. TRAM and TRIF: The Endosome Specific Adaptor Molecules

Metadatabase searches led to the discovery of TRAM (Figure 2) [30]. Similar to the TIRAP and MyD88 pair, TRAM is required for TRIF adaptor engagement [22]. In contrast to TIRAP, TRAM is myristoylated at its N-terminus [31] (Figure 3), allowing anchoring to the endosomal membrane. Mutations abolishing TRAM myristoylation restrain TRAM cytoplasmic localization, thereby inhibiting TRIF-generated signal transduction by TLR4 [31]. In addition, cell treatment with dynasore, a dynamin 2 guanosine triphosphate (GTPase) pharmacological inhibitor, demonstrated that TLR4 internalization mediates TRAM/TRIF signaling [22, 32].

Notably, TRIF is involved in antiviral protection by promoting the secretion of antiviral-specialized cytokines, namely, type I Interferons (IFN I : IFN-*α* and IFN-*β*) [33]. TRIF is recruited downstream of endosomal TLRs recognizing nucleic acids. This specific endosomal location, along with nucleic acid specific binding, protects from host DNA or mRNA detection-mediated autoimmunity. Indeed, lipofected host DNA stimulates TLR9 [34]. Viral carbohydrate and lipid structures are very similar to those observed in host cells and therefore do not represent suitable PAMPs. Instead, the immune system has evolved to express PRRs—TLRs included—that recognize viral nucleic acids [35]. For instance, TLR3 recognizes double-stranded RNA (dsRNA), and TLR9 binds unmethylated cytosine-phosphate-guanine (CpG) dinucleotides [36, 37]. Of note, *Trif*-deficient mice show impaired IFN-*β* expression in response to TLR3 and TLR4 ligands [38]. Thus, even if TLR4 detects bacterial MAMPs at the cell membrane, TLR4 induces antiviral pathways when localized within endosomes [28]. In 2014, more than ten years after this discovery, a similar function has been detailed regarding TLR2 [39].

## 3. TLR2 and TLR4: The “Only” TIRAP-Dependent TLRs

Signaling through TLR4, the most investigated TLR, has been well dissected in comparison to other TLRs (Figure 1). MyD88, the first identified TLR adaptor downstream of TLR4 [14], is considered to be the adaptor “of choice” for other TLRs, except TLR3. However, this MyD88-only assumption has been challenged by various studies, suggesting that TIRAP- and TRAM-dependent signaling may be used in a larger set of TLRs. In 2002, two works have revealed that TIRAP is specific to TLR2 and TLR4 [24, 40]. Recent reviews still mention these two papers [41–43]. In this section, we explore and contextualize those two hyperreferenced studies.

**Figure 4:**
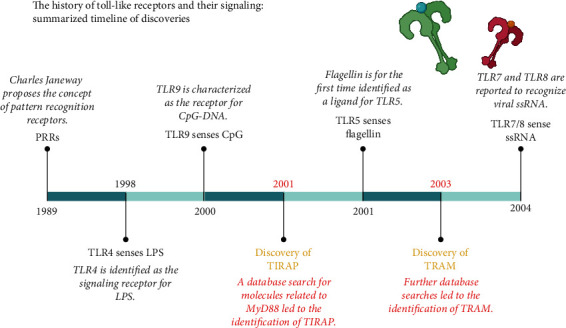
The history of Toll-like receptors and their signaling: summarized timeline of discoveries. Abbreviations: CpG-DNA: cytosine-phosphate-guanine-deoxyribo-nucleic acid; LPS: lipopolysaccharide; MyD88: myeloid differentiation primary response 88; PRRs: pattern recognition receptors; ssRNA: single-stranded ribonucleic acid; TIRAP: Toll/interleukin-1 receptor domain-containing adaptor protein; TLR4/5/7/8/9: Toll-like receptor 4/5/7/8/9; TRAM: TRIF-related adaptor molecule.

In 2001, flagellin was identified as a ligand for TLR5 (Figure 4). A year later, it was revealed that *Tirap*-deficient mice are still responsive to flagellin, implying that TLR5 signaling is TIRAP-independent [24]. However, TLR5 is not the only flagellin sensor. Indeed, NOD-like receptor caspase activation and recruitment domain-containing protein 4 (NLRC4 inflammasome) is very sensitive to flagellin [44] in the cytosol of myeloid cells [45]. Unfortunately, no cellular assessment was done to discriminate this in 2002, five years before the discovery that another sensor may play a compensatory role [24]. Since then, the idea that TLR5 signaling is TIRAP-independent became the norm.

In 2002, it was demonstrated that NF-*κ*B and MAPK induction downstream of TLR9 was TIRAP-independent [24]. It was shown that *Tirap*-deficient macrophages activate NF-*κ*B with delayed kinetics in response to TLR2 and TLR4 agonists, but not CpG [24]. Nevertheless, according to the NF-*κ*B assay, TLR2- and TLR4-mediated activations are fast (20 and 10 minutes, respectively), while NF-*κ*B activation does not start before 60-minute downstream of TLR9. No intermediary time was tested to exactly evaluate if the kinetics in response to CpG is delayed or not. MAPK assays were then performed [24]. MAPKs are not activated before one hour of CpG stimulation, but the phosphorylation status of JNK and p38 is reduced downstream of TLR9 in *Tirap*-deficient macrophages compared with wild-type macrophages. Unfortunately, these results were not taken into consideration by the authors. Subsequently, TLR9 gained its “directly binds MyD88” notoriety [46]. Additional studies would have been needed to further our understanding of the TLR9-induced signaling through other adaptors (Figure 1).

All these discoveries emerged during a very frenetic period (Figure 4) where information appeared as things progressed, without the supporting data to have a better view of TLR signaling. TLR4- and MyD88-related studies were particularly favored (Figure 1).

## 4. TLR5, TLR7, TLR8, and TLR9 Are Not “Only MyD88-Dependent” TLRs

### 4.1. TLR5

TLR5 is plasma membrane-localized and recognizes flagellin from invasive motile bacteria [47]. It has been well-described that TLR5 is only Myd88-dependent for its downstream signaling [48]. Yet, Choi et al. have demonstrated that TLR5 does require TIRAP to induce NF-*κ*B-dependent responses. Indeed, reduced *Tirap* gene expression in cultured colonocytes impaired the response to flagellin. Further immunoprecipitation experiments confirmed direct interaction between TLR5 and TIRAP, following flagellin exposure [49]. Of note, colonocytes represent a more relevant experimental model for TLR5 signaling studies, since they barely express NLRC4 [50]. Intriguingly, flagellin has been recently found to mediate IFN-*β* production in macrophages, after TLR5 internalization from the plasma membrane to endosomes [51]. In addition, TLR5 signaling was shown to be TRIF-dependent in human colonic cells [52]. TRIF directly interacts with TLR5 upon flagellin stimulation in NCM460 colonic cells [52]. In this study, TRAM also directly interacts with TLR5 in nonstimulated conditions, as opposed to TRIF. Unfortunately, this result was not taken into consideration by the authors, and more studies are required to elucidate such observation. Altogether, adaptors other than MyD88, including TIRAP and TRIF, may contribute to the induction of TLR5 downstream signaling.

### 4.2. TLR7 and TLR8

TLR7 and TLR8 are endosomal TLRs recognizing ssRNA, the reason why both are often represented together in the endosomal compartment. Additionally, TLR7 and TLR8 share common synthetic agonists, such as R-8748 (resiquimod) [53] or gardiquimod [54]. Even though they are localized in endosomes, TLR7 and TLR8 activate NF-*κ*B and IFN I-generating pathways [55]. In 2015, by using a peptide (decoy peptide 2R9) that blocks TIRAP recruitment, Piao et al. have shown that TLR7- and TLR8-dependent NF-*κ*B activations are TIRAP-dependent in macrophages [56]. More recently, experiments on Carp Toll-like receptor 8 (Tlr8) have disclosed that TLR8 can directly interact with the TIRAP adaptor and that such interaction is necessary for MyD88-dependent responses [57]. Regrettably, few researches have been done on TLR8 (Figure 1), and therefore, functional studies on mammalian TIRAP and TLR8 interactions are still lacking.

Interestingly, TRIF engagement and IFN secretion by TLR7 require another adaptor protein, TRAM [58]. Indeed, while *Tram*-deficient macrophages exhibit a complete NF-*κ*B response to the TLR7 ligand imiquimod, IFN I secretion is abolished. Whether TRAM may also play a role in TLR8 transduction has never been investigated. A potential TRAM compensatory role may explain why, in 2002, there was no impaired proliferation of *Tirap*-deficient splenocytes to R-848 [24]. But TRAM was discovered a year later (Figure 4), and such possibility could even not be suggested. Furthermore, downstream of TLRs, MyD88 activation, is required for cell division [59], which could explain why *MyD88*-deficient splenocytes show impaired proliferation to R-848 [24]. Taken together, these recent data suggest that, in addition to the known role of Myd88, TIRAP and TRAM can be involved in TLR7 and TLR8 signaling. However, one question remains: how endosomal TLR7 and TLR8 could activate both MyD88-dependent and -independent pathways? The beginning of an answer is provided by studies on TLR9 detailed as follows.

### 4.3. TLR9

TLR9 is an endosomal TLR that detects unmethylated CpG dinucleotides from viral and bacterial DNA [60] and is located in endosomes. TLR9 activation triggers NF-*κ*B and IFN I signaling pathways [61]. TIRAP expression enhances the MyD88-dependent response mediated by TLR9 [62]. Moreover, immortalized bone marrow-derived macrophages (iBMDMs) isolated from *Tirap* KO (knockout) mice do not respond to ODN1668, a TLR9 agonist [56]. iBMDMs represent a useful experimental model to explore signaling, as they retain the signaling properties of primary macrophages [62]. TLR9-provoked secretion of TNF-*α* and IL-6 is TIRAP-dependent in iBMDMs [56]. In this study, these data were confirmed by using a decoy peptide that directly targets TIRAP. More recently, the same group has demonstrated that ODN-induced cytokine secretion and lethality are abrogated by intraperitoneally pretreating mice with the 9R34 decoy peptide, a more specific TLR9 inhibitor [63]. Finally, *Tirap*-deficient macrophages infected with herpes virus simplex (HSV), a natural TLR9 activator, are unable to trigger NF-*κ*B signaling [62].

The use of two structurally diverse synthetic TLR9 ligands uncovers surprising outcomes. Plasmacytoid dendritic cells do express TLR7 and TLR9 within endosomal compartments [64], which allow these cells to produce high amounts of IFN type I, in contrast to conventional dendritic cells [65]. CpG-A treatment led to increased IFN I production in mouse plasmacytoid dendritic cells, while proinflammatory cytokine release, related to NF-*κ*B activation, was induced only in response to CpG-B [66]. The authors explained their data through distinct localization of both ligands, since CpG-A and CpG-B do not always traffic in the same way within cells. For instance, in plasmacytoid dendritic cells, CpG-A is retained in early endosomes, whereas CpG-B translocates to late endosomes and lysosomes [67].

Dendritic cells are professional antigen-presenting cells, also acting as mediators between the innate and the adaptative immune systems [68]. Primary dendritic cells, namely, bone marrow-derived dendritic cells (BMDCs), represent an interesting working model, as primary BMDC cultures can be matured in a number of cell types, including dendritic cells and macrophages [69]. In BMDCs, CpG-A and CpG-B ligands are both transported to late endosomes and lysosomes, leading to NF-*κ*B responses. In addition, conventional dendritic cells produce IFN I when stimulated with dioleoyl-3-trimethyl-ammonium propane- (DOTAP-) lipofected CpG-A, which is retained in endosomes [67]. PI(3,5)P_2_, abundant in late endosomes and lysosomes [70], facilitates the anchoring of TIRAP in response to CpG [71]. In plasmacytoid dendritic cells, TLR9 stimulation initiates IFN I expression in a TRIF-dependent manner [72], which was recently confirmed in macrophages [73]. But studies on the role of TRAM in endosome-mediated TLR9 responses are still missing. Taken together, these data support a model in which TLR9 functionally traffics within the cell to trigger distinct pathways, by recruiting different signaling adaptors other than Myd88, with differences related to the cell type. Most of the research on TLR9 has been done in plasmacytoid dendritic cells, specialized in antiviral responses [65]. It would be relevant to see if similar results can be observed in other cell types.

## 5. Why the Endosomal Trio TLR7/8/9 Is Believed to Be MyD88-Dependent

As opposed to IRF3, the *IFN-beta*-specialized transcription factor, IRF7, is less specific and promotes both *IFN-alpha* and *IFN-beta* transcriptions [74]. Like IRF3, IRF7 is phosphorylated by members of the I*κ*B kinase family (IKKs), including IKK*α*, IKK*β*, IKK*ε*, and TRAF-associated NF-*κ*B activator-binding kinase 1 (TBK1) [75]. IKK*α* and IKK*β* are also involved in the canonical NF-*κ*B pathway. Despite redundancy, IKK*ε* and TBK1 are the two more specialized kinases in IRF3 phosphorylation-promoted activation [76]. Some papers have provided explanations for this preference. Indeed, IRF3 regulation requires phosphatidylinositol-5-phosphate (PI5P) [71]. PI5P, which is enriched in membranes of early endosomes during viral infection, binds to both IRF3 and TBK1 to facilitate complex formation [71]. TRAM, involved in TBK1 and IRF3 activations, binds phosphatidylinositol-3-phosphate (PIP3) and PI5P [22] and is localized to early endosomes as well. Findings on phosphoinositide-mediated effector recruitment in TLR signaling are summarized in Table 1. IRF7 interacts with MyD88 and TRAF6 [77], required for IKK engagement and IKK*α*- and IKK*β*-induced IRF7 phosphorylation [75]. IRF7 is thus activated downstream of TLR7, TLR8, and TLR9, all recognized to “directly bind MyD88.”

Very recently, it has been demonstrated that TIRAP is also necessary for IRF7 phosphorylation in macrophages and human plasmacytoid dendritic cells, by bridging MyD88 to TLR7 [79]. Whether TLR8 and TLR9 adopt a similar requirement for TIRAP to activate IRF7 remains to be determined. Of note, in plasmacytoid dendritic cells, the TLR9 agonist CpG-A, initiating IFN I release, colocalizes with IRF7 in early endosomes [66]. TIRAP binds to PI(4,5)P_2_ at the cytoplasmic membrane and to PI3P on early endosomes [17, 27, 78]. Thus, while IRF7 activation is MyD88-dependent, some recent data suggest that TIRAP may be needed for such activation.

## 6. An Emerging Model for TLR/TIRAP/MyD88 Signaling

**Figure 5:**
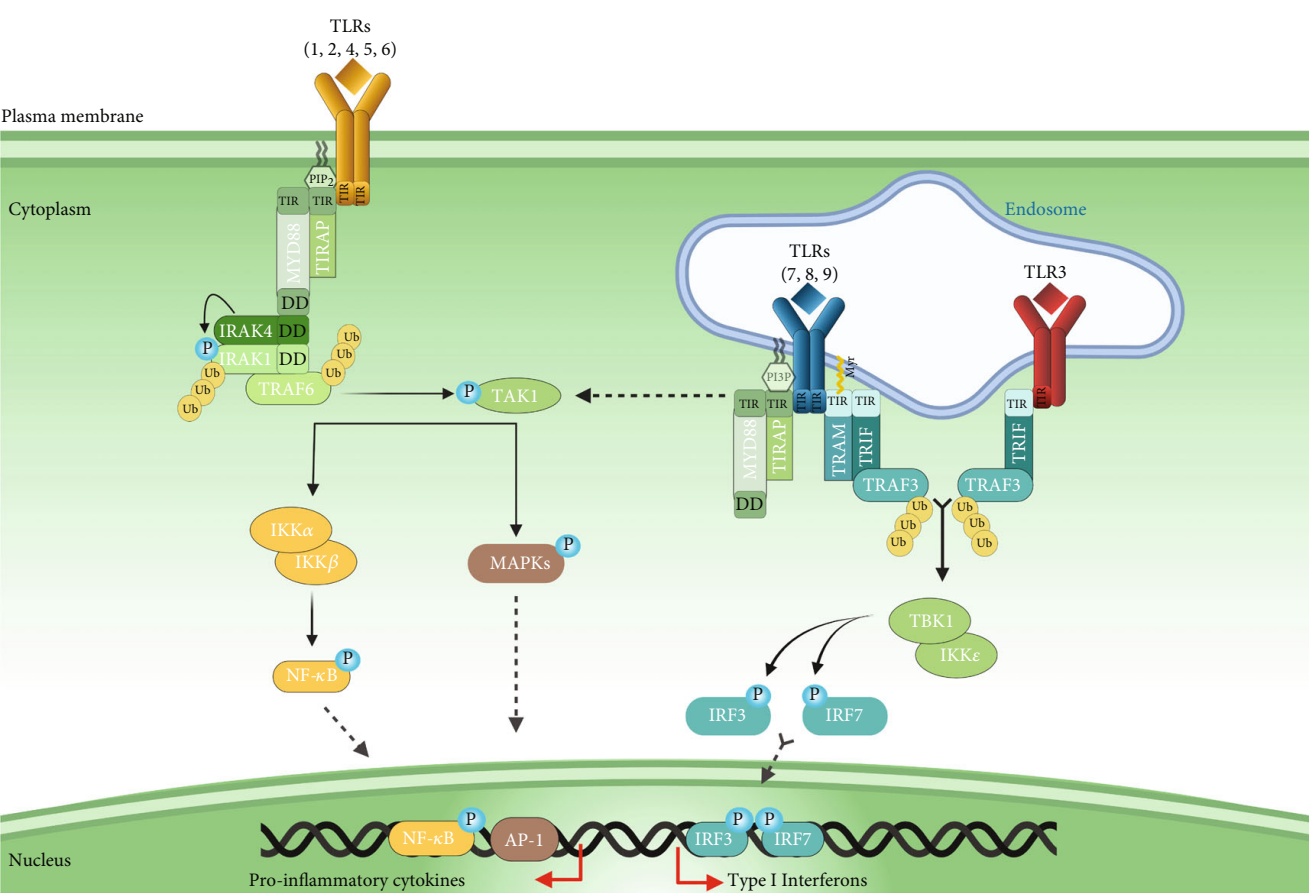
Emerging model for TLR signaling. Recent data suggest a new model according to which all TLRs, but TLR3, are TIRAP-dependent for MyD88-mediated pathways and TRAM-dependent for the TRIF cascade. TLR3 directly recruits the TRIF adaptor to the endosomal compartment. TRAF3: TNF receptor-associated factor 3.

According to an emerging TLR signaling model (Figure 5), all TLRs except TLR3 are TIRAP-dependent for MyD88-mediated pathways [80]. However, one question remains: what is the biological relevance of the TIRAP bridging adaptor, knowing that all TLRs can directly bind MyD88 through TIR-TIR interactions? Answers are provided by crystallographic structural studies and by the myddosome discovery [81, 82]. The myddosome is a multiproteic and functional signaling complex, including six MyD88, four IRAK4, and four IRAK1 subunits [41, 80], triggering NF-*κ*B activation. 3D structures reveal that each TLR4 homodimer recruits two TIRAP homodimers, each recruiting in turn four MyD88 molecules [80]. So, eight MyD88 molecules are clustered following TLR4 homodimerization, which is enough to engage a myddosome. By amplifying MyD88 engagement, TIRAP allows transduction of favorable signal downstream of TLRs. This TLR4-dependent pattern may be valid for all TLRs except TLR3, according to the authors [80] and discussed in a recent review [83].

## 7. Clinical Relevance

### 7.1. *TIRAP* Gene Polymorphisms and Pathogenesis

TLR receptors evolved before the adaptive immune system to form an indispensable first line of innate defense [84]. TLRs play key roles in homeostatic as well as in pathogenic responses in many disease settings. TLR signaling represents an important target for putative treatments. As we mentioned before, TIRAP and TRAM are essential TLR bridging adaptors, while largely neglected in the scientific literature, as opposed to Myd88 (Figure 1). Remarkably, small nucleotide polymorphisms (SNPs) of TLRs and their adaptors are associated with infections and other diseases, such as atherosclerosis, asthma, or colorectal cancer [85]. Notably, TIRAP is the most polymorphic of all adaptors, harboring at least eight nonsynonymous mutations in its coding sequence [86]. Some reported *TIRAP* gene SNPs are presented in Table 2. Excluding the roles of TIRAP and other adaptors in TLR responses reduces our capacity to fully comprehend the TLR-dependent regulatory mechanisms implicated in acute and chronic disorders.

Interestingly, the *TIRAP* gene S180L SNP is associated with protection against infections and autoimmune diseases, such as invasive pneumococcal disease, malaria, and systemic lupus erythematosus [88, 93]. The chronic Chagas cardiomyopathy is a tropical parasitic disease caused by the intracellular protozoan *Trypanosoma cruzi* [94], detected by TLR4 and TLR2/6 [95]. Up to 45% of patients with chronic infections develop cardiomyopathy, between 10 and 30 years after the initial sickness [94]. It has been reported that heterozygosity for the *TIRAP* S180L variant is associated with lower risk of developing chronic Chagas cardiomyopathy [91]. Mechanistically, the authors propose that the S180L variant leads to decreased signal transduction downstream of TLR2 and TLR4. Accordingly, *Tirap*-deficient MEFs, transfected with a plasmid encoding *Tirap* L180, failed to induce the NF-*κ*B pathway [93]. In contrast, homozygosity for the S180L variant confers increased susceptibility to invasive pneumococcal disease, while the heterozygosity state provides a protective phenotype [93]. The authors speculate that S180L homozygosity results in decreased NF-*κ*B signaling, thus aggravating susceptibility to infections.

The TIRAP D96N variant is considered a loss-of-function SNP [88]. Crystal structure of TIRAP reveals that amino acids D96 and S180 are within the TIR domain interacting with the MyD88 adaptor protein [96]. A worldwide polymorphism distribution investigation proposes that the TIRAP variant S180L has been evolutionary selected to provide protection against septic shock [97]. This study supplies a world map of S180L distribution, which intriguingly correlates negatively with global sepsis incidence [98]. Knowing that all TLRs are involved in septic shock [99], these data imply that the role of TIRAP in TLR signaling related to human diseases should be better considered. Recent data by Rajpoot et al. provide new structural studies and insights on TIRAP [100]. Using an *in silico* approach, they have determined that the phospho-motif P-Y86 on TIRAP interacts with p38 MAPK for activation, which is worth to be validated in an *in vitro* model [101]. Activated p38 is a well-described proinflammatory mediator involved in acute and chronic inflammations [102]. Rajpoot et al. have also identified new TIRAP inhibitors by combining several docking tools, and their future validation may lead to novel treatments against inflammatory disorders [103]. These promising docking designs may well promote further research on the TIRAP adaptor.

### 7.2. *TRAM* Gene Polymorphisms and Tuberculosis

Unfortunately, few studies have reported *TRAM* (also named *TICAM2* for TIR domain-containing adaptor molecule 2) gene polymorphisms, since TRAM is the less investigated TLR-related adaptor (Figure 1). In 2015, one polymorphism localized in the flanking 5′ untranslated region (UTR) of *TRAM* was associated with tuberculosis caused by the bacteria *Mycobacterium tuberculosis* [104, 105]. Different components of *Mycobacterium tuberculosis* interact with TLRs (e.g., TLR2, TLR4, TLR8 and TLR9) in macrophages, natural killer (NK) cells, dendritic cells and T cells and and induce an appropriate immune response to overcome infection [106]. While the significance of *TRAM* polymorphism and how it relates to its expression are unknown, these observations point to a link between TRAM and tuberculosis infection. Interestingly, levels of TRAM expression in peripheral blood mononuclear cells (PBMCs) predict with 80% accuracy whether subjects are high or low responders to a poxvirus vector tuberculosis vaccine candidate, expressing antigen 85A [107].

In BMDMs, the heat shock protein 70 (Hsp70) is derived from *Mycobacterium tuberculosis* signals through TLR2 and TLR4 and the TIRAP, MyD88, TRAM, and TRIF adaptor molecules [108]. More studies are needed to understand the role of TRAM adaptor in tuberculosis infection and more largely in human chronic diseases.

### 7.3. Coronavirus Disease 2019 (COVID-19)

We are facing new sanitary challenges with COVID-19, the most recent coronavirus-mediated acute respiratory illness caused by the SARS-coronavirus-2 (SARS-CoV-2). Since this viral infection causes severe symptoms through the induction of a cytokine storm, many groups have studied TLR signaling to identify therapeutic targets. Prior SARS-CoV-1 research has exposed the importance of TLR adaptors in viral responses. For example, overexpression of the SARS-CoV-1 membrane protein (M) in HEK293T cells leads to increased TIRAP and TRAM protein levels in comparison to control cells. This correlates with upregulated IFN-b- and NF-*κ*B-related gene expressions [109]. *Tram^−/−^* mice are more susceptible to mouse-adapted SARS-CoV-1 infection, without extra mortality [110]. Genetic studies in mice have revealed *Tram* as a susceptibility gene for SARS-CoV-1 infection [111], underlining the importance of IFN I release during SARS-CoV-1 infection recovery. In line with this observation, decreased aging-associated number of plasmacytoid dendritic cells is associated with COVID-19 severity [112]. In addition, neutralizing autoantibodies against IFN I have been detected in patients with life-threatening COVID-19 [113]. Finally, increased TIRAP phosphorylation is detected in COVID-19-infected individuals [114]. These data suggest that both TIRAP and TRAM adaptors play a role in the control of SARS-CoV-2 infections.

The above results highlight the importance to study TLR signaling and to include TLR adaptor regulatory functions to understand COVID-19 disease. The SARS-CoV genomes activate TLR7 [115]. Rare putative loss-of-function variants of the X-chromosome-located *TLR7* gene are associated with altered type I IFN expression in young men with severe COVID-19 [116]. TLR8, being more specific, recognizes both SARS-CoV-2 ssRNA and derived ribonuclease T2 degradation products [117]. Thus, these recent findings call for more research on TLR7 and TLR8 (Figure 1), as targets of SARS-CoV-2 viral motifs. Clinical trials aimed to stimulate endosomal TLRs to promote IFN I production at the early steps of infection or to inhibit TLRs to reduce the NF-*κ*B-promoted cytokine storm are ongoing. Imiquimod, a TLR7 ligand, has been proposed as an option to manage the initial stages of COVID-19 [118, 119]. Conversely, clinical studies exploring TLR blockade during COVID-19 late steps are ongoing. MERCK KGaA has initiated a randomized double-blind phase II clinical trial with M5049, a selective TLR7/8 pharmacological inhibitor initially designed to treat autoimmunity [120], for the treatment of severe symptoms of COVID-19 [121].

## 8. Conclusion

In this review, we have underscored the importance of TIRAP and TRAM bridging molecules in MyD88 and TRIF recruitments. In the last few years, most research was performed on TLR4 because of the importance of its ligand LPS [122] in mediating sepsis, a worldwide public health issue [123]. Sepsis is indeed the leading cause of death in intensive care units in the United States [124]. Gram-bacterial sepsis mortality is 20 to 50% among total sepsis deaths [125]. In 2010, Chaby reported that a paper on LPS was published every two hours [123]. Therefore, TLR4 has been extensively explored in comparison to other TLRs, and studies about TLR4 signaling have been fundamental in discovering the TIRAP-MyD88 and TRAM-TRIF signaling patterns. Unexpectedly, these patterns were also revealed downstream of TLR2 [39]. Pursuing such efforts to analyze other TLRs is needed to discover treatments against novel infections, such as COVID-19. Thus, while TLR signaling is believed to be “well-described,” further studies are warranted for a complete understanding of TLR signaling pathways, including the role of TIRAP and TRAM adaptors.

## Figures and Tables

**Table 1:** Cellular compartments for TLR signaling effector recruitment.

TLR effector	Related pathway	TLR	PI lipids	Cellular compartments	Cell types	References
TIRAP	NF-*κ*B	TLR2, TLR4	PI(4,5)P_2_	Plasma membrane	Human monocytes, macrophages	[28, 78]
		TLR4, TLR9	PI(3,5)P_2_	Lysosome	BMDMs	[62, 67]
	IFN I (*via* IRF7)	TLR4	PI3P, PI5P	Early endosome	MEFs, macrophages	[17, 27]
TRAM	IFN I (via TRIF)				Macrophages	[22]
TBK1		TLR3, TLR4	PI5P		MEFs, GMDCs	[71]
IRF3						

Abbreviations: BMDMs: bone marrow-derived macrophages; GMDCs: genetically modified dendritic cells; IFN I: type I interferons; IRF3: interferon regulatory factor 3; IRF7: interferon regulatory factor 7; MEFs: mouse embryonic fibroblasts; NF-*κ*B: nuclear factor-*κ*B; PI: phosphatidylinositol; PI3P: phosphatidylinositol-3-phosphate; PI5P: phosphatidylinositol-5-phosphate; PI(3,5)P_2_: phosphatidylinositol-3,5-biphosphate; PI(4,5)P_2_: phosphatidylinositol-4,5-biphosphate; TBK1: TRAF-associated NF-*κ*B activator-binding kinase 1; TIRAP: Toll/interleukin-1 receptor domain-containing adaptor protein; TLR: Toll-like receptor; TRAM: TRIF-related adaptor molecule; TRIF: TIR domain-containing adaptor-inducing interferon-*β*.

**Table 2:** Reported SNPs in the *TIRAP* gene.

SNP	Associated diseases	References
S55N	Meningeal tuberculosis	[87]
D96N	Lymphoma	[88]
E132K	Atopic dermatitis	[89]
S180L	Malaria, sepsis, and Chagas cardiomyopathy	[89–91]
C539T	Tuberculosis susceptibility	[92]

Abbreviations: C: cysteine; D: aspartate; E: glutamate; K: lysine; L: leucine; N: asparagine; S: serine; SNP: single-nucleotide polymorphism; T: threonine; TIRAP: Toll/interleukin-1 receptor domain-containing adaptor protein.

## References

[1] Lemaitre B., Nicolas E., Michaut L., Reichhart J. M., Hoffmann J. A. (1996). The dorsoventral regulatory gene cassette spätzle/Toll/cactus controls the potent antifungal response in Drosophila adults. *Cell*.

[2] Medzhitov R., Preston-Hurlburt P., Janeway C. A. (1997). A human homologue of the Drosophila Toll protein signals activation of adaptive immunity. *Nature*.

[3] Du X., Poltorak A., Wei Y., Beutler B. (2000). Three novel mammalian toll-like receptors: gene structure, expression, and evolution. *European Cytokine Network*.

[4] Beutler B., Jiang Z., Georgel P. (2006). Genetic analysis of host resistance: Toll-like receptor signaling and immunity at large. *Annual Review of Immunology*.

[5] Matsushima N., Tanaka T., Enkhbayar P. (2007). Comparative sequence analysis of leucine-rich repeats (LRRs) within vertebrate toll-like receptors. *BMC Genomics*.

[6] Delneste Y., Beauvillain C., Jeannin P. (2007). Innate immunity: structure and function of TLRs. *Medical Science (Paris)*.

[7] Takeda K., Kaisho T., Akira S. (2003). Toll-like receptors. *Annual Review of Immunology*.

[8] Mukherjee S., Karmakar S., Babu S. P. S. (2016). TLR2 and TLR4 mediated host immune responses in major infectious diseases: a review. *The Brazilian Journal of Infectious Diseases*.

[9] Heil F., Ahmad-Nejad P., Hemmi H. (2003). The Toll-like receptor 7 (TLR7)-specific stimulus loxoribine uncovers a strong relationship within the TLR7, 8 and 9 subfamily. *European Journal of Immunology*.

[10] Latz E., Schoenemeyer A., Visintin A. (2004). TLR9 signals after translocating from the ER to CpG DNA in the lysosome. *Nature Immunology*.

[11] Matsumoto M., Funami K., Tanabe M. (2003). Subcellular localization of Toll-like receptor 3 in human dendritic cells. *Journal of Immunology*.

[12] Lund J. M., Alexopoulou L., Sato A. (2004). Recognition of single-stranded RNA viruses by Toll-like receptor 7. *Proceedings of the National Academy of Sciences of the United States of America*.

[13] Saitoh S., Akashi S., Yamada T. (2004). Lipid A antagonist, lipid IVa, is distinct from lipid A in interaction with Toll-like receptor 4 (TLR4)-MD-2 and ligand-induced TLR4 oligomerization. *International Immunology*.

[14] Kawai T., Adachi O., Ogawa T., Takeda K., Akira S. (1999). Unresponsiveness of MyD88-deficient mice to endotoxin. *Immunity*.

[15] Krishnan J., Selvarajoo K., Tsuchiya M., Lee G., Choi S. (2007). Toll-like receptor signal transduction. *Experimental & Molecular Medicine*.

[16] Takeuchi O., Takeda K., Hoshino K., Adachi O., Ogawa T., Akira S. (2000). Cellular responses to bacterial cell wall components are mediated through MyD88-dependent signaling cascades. *International Immunology*.

[17] Patra M. C., Choi S. (2018). Insight into phosphatidylinositol-dependent membrane localization of the innate immune adaptor protein Toll/interleukin 1 receptor domain-containing adaptor protein. *Frontiers in Immunology*.

[18] Bulut Y., Faure E., Thomas L., Equils O., Arditi M. (2001). Cooperation of toll-like receptor 2 and 6 for cellular activation by soluble tuberculosis factor and Borrelia burgdorferi outer surface protein A lipoprotein: role of Toll-interacting protein and IL-1 receptor signaling molecules in Toll-like receptor 2 signaling. *Journal of Immunology*.

[19] Yamamoto M., Okamoto T., Takeda K. (2006). Key function for the Ubc13 E2 ubiquitin-conjugating enzyme in immune receptor signaling. *Nature Immunology*.

[20] Mansell A., Smith R., Doyle S. L. (2006). Suppressor of cytokine signaling 1 negatively regulates toll-like receptor signaling by mediating Mal degradation. *Nature Immunology*.

[21] Mansell A., Brint E., Gould J. A., O’Neill L. A., Hertzog P. J. (2004). Mal interacts with tumor necrosis factor receptor-associated factor (TRAF)-6 to mediate NF-kappaB activation by toll-like receptor (TLR)-2 and TLR4. *The Journal of Biological Chemistry*.

[22] Kagan J. C., Su T., Horng T., Chow A., Akira S., Medzhitov R. (2008). TRAM couples endocytosis of Toll-like receptor 4 to the induction of interferon-*β*. *Nature Immunology*.

[23] Horng T., Barton G. M., Medzhitov R. (2001). TIRAP: an adapter molecule in the Toll signaling pathway. *Nature Immunology*.

[24] Horng T., Barton G. M., Flavell R. A., Medzhitov R. (2002). The adaptor molecule TIRAP provides signalling specificity for Toll-like receptors. *Nature*.

[25] Fitzgerald K. A., Palsson-McDermott E. M., Bowie A. G. (2001). Mal (MyD88-adapter-like) is required for Toll-like receptor-4 signal transduction. *Nature*.

[26] Lin S.-C., Lo Y.-C., Wu H. (2010). Helical assembly in the MyD88-IRAK4-IRAK2 complex in TLR/IL-1R signalling. *Nature*.

[27] Kagan J. C., Medzhitov R. (2006). Phosphoinositide-mediated adaptor recruitment controls Toll-like receptor signaling. *Cell*.

[28] Tanimura N., Saitoh S., Matsumoto F., Akashi-Takamura S., Miyake K. (2008). Roles for LPS-dependent interaction and relocation of TLR4 and TRAM in TRIF- signaling. *Biochemical and Biophysical Research Communications*.

[29] Murphy J. E., Padilla B. E., Hasdemir B., Cottrell G. S., Bunnett N. W. (2009). Endosomes: a legitimate platform for the signaling train. *Proceedings of the National Academy of Sciences of the United States of America*.

[30] Fitzgerald K. A., Rowe D. C., Barnes B. J. (2003). LPS-TLR4 signaling to IRF-3/7 and NF-kappaB involves the toll adapters TRAM and TRIF. *The Journal of Experimental Medicine*.

[31] Rowe D. C., McGettrick A. F., Latz E. (2006). The myristoylation of TRIF-related adaptor molecule is essential for Toll-like receptor 4 signal transduction. *Proceedings of the National Academy of Sciences of the United States of America*.

[32] Kirchhausen T., Macia E., Pelish H. E. (2008). Use of dynasore, the small molecule inhibitor of dynamin, in the regulation of endocytosis. *Methods in Enzymology*.

[33] Guo B., Cheng G. (2007). Modulation of the interferon antiviral response by the TBK1/IKKi adaptor protein TANK. *The Journal of Biological Chemistry*.

[34] Yasuda K., Rutz M., Schlatter B. (2006). CpG motif-independent activation of TLR9 upon endosomal translocation of “natural” phosphodiester DNA. *European Journal of Immunology*.

[35] Diebold S. S., Lombardi G., Riffo-Vasquez Y. (2009). Activation of Dendritic Cells by Toll-Like Receptors and C-Type Lectins. *Dendritic Cells*.

[36] Hemmi H., Takeuchi O., Kawai T. (2000). A Toll-like receptor recognizes bacterial DNA. *Nature*.

[37] Bauer S., Kirschning C. J., Häcker H. (2001). Human TLR9 confers responsiveness to bacterial DNA via species-specific CpG motif recognition. *Proceedings of the National Academy of Sciences of the United States of America*.

[38] Yamamoto M., Sato S., Hemmi H. (2003). Role of adaptor TRIF in the MyD88-independent toll-like receptor signaling pathway. *Science*.

[39] Stack J., Doyle S. L., Connolly D. J. (2014). TRAM is required for TLR2 endosomal signaling to type I IFN induction. *Journal of Immunology*.

[40] Yamamoto M., Sato S., Hemmi H. (2002). Essential role for TIRAP in activation of the signalling cascade shared by TLR2 and TLR4. *Nature*.

[41] Balka K. R., De Nardo D. (2019). Understanding early TLR signaling through the myddosome. *Journal of Leukocyte Biology*.

[42] Javaid N., Yasmeen F., Choi S. (2019). Toll-like receptors and relevant emerging therapeutics with reference to delivery methods. *Pharmaceutics*.

[43] Fitzgerald K. A., Kagan J. C. (2020). Toll-like receptors and the control of immunity. *Cell*.

[44] Miao E. A., Andersen-Nissen E., Warren S. E., Aderem A. (2007). TLR5 and Ipaf: dual sensors of bacterial flagellin in the innate immune system. *Seminars in Immunopathology*.

[45] Franchi L., Amer A., Body-Malapel M. (2006). Cytosolic flagellin requires Ipaf for activation of caspase-1 and interleukin 1*β* in salmonella-infected macrophages. *Nature Immunology*.

[46] Wagner H. (2004). The immunobiology of the TLR9 subfamily. *Trends in Immunology*.

[47] Hayashi F., Smith K. D., Ozinsky A. (2001). The innate immune response to bacterial flagellin is mediated by Toll-like receptor 5. *Nature*.

[48] Gewirtz A. T., Navas T. A., Lyons S., Godowski P. J., Madara J. L. (2001). Cutting edge: bacterial flagellin activates basolaterally expressed TLR5 to induce epithelial proinflammatory gene expression. *Journal of Immunology*.

[49] Choi Y. J., Jung J., Chung H. K., Im E., Rhee S. H. (2013). PTEN regulates TLR5-induced intestinal inflammation by controlling Mal/TIRAP recruitment. *The FASEB Journal*.

[50] Tissue expression of NLRC4- summary-The Human Protein Atlas. https://www.proteinatlas.org/ENSG00000091106-NLRC4/tissue.

[51] Kang W., Park A., Huh J. W. (2020). Flagellin-stimulated production of interferon-*β* promotes anti-flagellin IgG2c and IgA responses. *Molecules and Cells*.

[52] Choi Y. J., Im E., Chung H. K., Pothoulakis C., Rhee S. H. (2010). TRIF mediates Toll-like receptor 5-induced signaling in intestinal epithelial cells. *The Journal of Biological Chemistry*.

[53] Hurst J., Prinz N., Lorenz M. (2009). TLR7 and TLR8 ligands and antiphospholipid antibodies show synergistic effects on the induction of IL-1*β* and caspase-1 in monocytes and dendritic cells. *Immunobiology*.

[54] Buitendijk M., Eszterhas S. K., Howell A. L. (2013). Gardiquimod: a Toll-like receptor-7 agonist that inhibits HIV type 1 infection of human macrophages and activated T cells. *AIDS Research and Human Retroviruses*.

[55] Heil F., Hemmi H., Hochrein H. (2004). Species-specific recognition of single-stranded RNA via toll-like receptor 7 and 8. *Science*.

[56] Piao W., Shirey K. A., Ru L. W. (2015). A decoy peptide that disrupts TIRAP recruitment to TLRs is protective in a murine model of influenza. *Cell Reports*.

[57] Shan S., Liu R., Jiang L. (2018). Carp Toll-like receptor 8 (Tlr8): an intracellular Tlr that recruits TIRAP as adaptor and activates AP-1 pathway in immune response. *Fish & Shellfish Immunology*.

[58] Shevlin E., Miggin S. M. (2014). The TIR-domain containing adaptor TRAM is required for TLR7 mediated RANTES production. *PLoS One*.

[59] Chen L., Deng H., Cui H. (2018). Inflammatory responses and inflammation-associated diseases in organs. *Oncotarget*.

[60] Klinman D. M. (2006). Adjuvant activity of CpG oligodeoxynucleotides. *International Reviews of Immunology*.

[61] Martínez-Campos C., Burguete-García A. I., Madrid-Marina V. (2017). Role of TLR9 in oncogenic virus-produced cancer. *Viral Immunology*.

[62] Bonham K. S., Orzalli M. H., Hayashi K. (2014). A promiscuous lipid-binding protein diversifies the subcellular sites of toll- like receptor signal transduction. *Cell*.

[63] Javmen A., Szmacinski H., Lakowicz J. R., Toshchakov V. Y. (2018). Blocking TIR domain interactions in TLR9 signaling. *Journal of Immunology*.

[64] Gill M. A., Bajwa G., George T. A. (2010). Counterregulation between the Fc*ε*RI pathway and antiviral responses in human plasmacytoid dendritic cells. *Journal of Immunology*.

[65] Villadangos J. A., Young L. (2008). Antigen-presentation properties of plasmacytoid dendritic cells. *Immunity*.

[66] Honda K., Ohba Y., Yanai H. (2005). Spatiotemporal regulation of MyD88-IRF-7 signalling for robust type-I interferon induction. *Nature*.

[67] Oosenbrug T., van de Graaff M. J., Ressing M. E., van Kasteren S. I. (2017). Chemical tools for studying TLR signaling dynamics. *Cell Chemical Biology*.

[68] Monga I., Kaur K., Dhanda S. K. (2022). Revisiting hematopoiesis: applications of the bulk and single-cell transcriptomics dissecting transcriptional heterogeneity in hematopoietic stem cells. *Briefings in Functional Genomics*.

[69] Labeur M. S., Roters B., Pers B. (1999). Generation of tumor immunity by bone marrow-derived dendritic cells correlates with dendritic cell maturation stage. *Journal of Immunology*.

[70] Catimel B., Schieber C., Condron M. (2008). The PI(3,5)P2 and PI(4,5)P2 interactomes. *Journal of Proteome Research*.

[71] Kawasaki T., Takemura N., Standley D. M., Akira S., Kawai T. (2013). The second messenger phosphatidylinositol-5-phosphate facilitates antiviral innate immune signaling. *Cell Host & Microbe*.

[72] Volpi C., Fallarino F., Pallotta M. T. (2013). High doses of CpG oligodeoxynucleotides stimulate a tolerogenic TLR9-TRIF pathway. *Nature Communications*.

[73] Sauter I. P., Madrid K. G., de Assis J. B. (2019). TLR9/MyD88/TRIF signaling activates host immune inhibitory CD200 in *Leishmania* infection. *Insight*.

[74] Sato M., Hata N., Asagiri M., Nakaya T., Taniguchi T., Tanaka N. (1998). Positive feedback regulation of type I *IFN* genes by the IFN-inducible transcription factor IRF-7. *FEBS Letters*.

[75] Wang R.-P., Zhang M., Li Y. (2008). Differential regulation of IKK*α*-mediated activation of IRF3/7 by NIK. *Molecular Immunology*.

[76] Fitzgerald K. A., McWhirter S. M., Faia K. L. (2003). IKK*ε* and TBK1 are essential components of the IRF3 signaling pathway. *Nature Immunology*.

[77] Kawai T., Sato S., Ishii K. J. (2004). Interferon-*α* induction through Toll-like receptors involves a direct interaction of IRF7 with MyD88 and TRAF6. *Nature Immunology*.

[78] Marat A. L., Haucke V. (2016). Phosphatidylinositol 3-phosphates-at the interface between cell signalling and membrane traffic. *The EMBO Journal*.

[79] Leszczyńska E., Makuch E., Mitkiewicz M. (2020). Absence of Mal/TIRAP results in abrogated imidazoquinolinones-dependent activation of IRF7 and suppressed IFN*β* and IFN-I activated gene production. *International Journal of Molecular Sciences*.

[80] Guven-Maiorov E., Keskin O., Gursoy A. (2015). The architecture of the TIR domain signalosome in the Toll-like receptor-4 signaling pathway. *Scientific Reports*.

[81] Motshwene P. G., Moncrieffe M. C., Grossmann J. G. (2009). An oligomeric signaling platform formed by the Toll-like receptor signal transducers MyD88 and IRAK-4. *The Journal of Biological Chemistry*.

[82] Gay N. J., Gangloff M., O’Neill L. A. J. (2011). What the Myddosome structure tells us about the initiation of innate immunity. *Trends in Immunology*.

[83] Belhaouane I., Hoffmann E., Chamaillard M., Brodin P., Machelart A. (2020). Paradoxical roles of the MAL/Tirap adaptor in pathologies. *Frontiers in Immunology*.

[84] Gorvel J.-P. (2003). *Intracellular Pathogens in Membrane Interactions and Vacuole Biogenesis*.

[85] Lin Y.-T., Verma A., Hodgkinson C. P. (2012). Toll-like receptors and human disease: lessons from single nucleotide polymorphisms. *Current Genomics*.

[86] Nagpal K., Plantinga T. S., Wong J. (2009). A TIR domain variant of MyD88 adapter-like (Mal)/TIRAP results in loss of MyD88 binding and reduced TLR2/TLR4 signaling. *The Journal of Biological Chemistry*.

[87] Hawn T. R., Dunstan S. J., Thwaites G. E. (2006). A polymorphism in toll-interleukin 1 receptor domain containing adaptor protein is associated with susceptibility to meningeal tuberculosis. *The Journal of Infectious Diseases*.

[88] George J., Kubarenko A. V., Rautanen A. (2010). MyD88 adaptor-like D96N is a naturally occurring loss-of-function variant of *TIRAP*. *Journal of Immunology*.

[89] An Y., Ohnishi H., Matsui E. (2011). Genetic variations in MyD88 adaptor-like are associated with atopic dermatitis. *International Journal of Molecular Medicine*.

[90] Castiblanco J., Varela D.-C., Castaño-Rodríguez N., Rojas-Villarraga A., Hincapié M.-E., Anaya J.-M. (2008). TIRAP (MAL) S180L polymorphism is a common protective factor against developing tuberculosis and systemic lupus erythematosus. *Infection, Genetics and Evolution*.

[91] Ramasawmy R., Cunha-Neto E., Fae K. C. (2009). Heterozygosity for the S180L variant of *MAL*/*TIRAP*, a gene expressing an adaptor protein in the toll-like receptor pathway, is associated with lower risk of developing chronic Chagas cardiomyopathy. *The Journal of Infectious Diseases*.

[92] Liu Q., Li W., Li D., Feng Y., Tao C. (2014). TIRAP C539T polymorphism contributes to tuberculosis susceptibility: evidence from a meta-analysis. *Infection, Genetics and Evolution*.

[93] Khor C. C., Chapman S. J., Vannberg F. O. (2007). A Mal functional variant is associated with protection against invasive pneumococcal disease, bacteremia, malaria and tuberculosis. *Nature Genetics*.

[94] Pérez-Molina J. A., Molina I. (2018). Chagas disease. *Lancet*.

[95] Rodrigues M. M., Oliveira A. C., Bellio M. (2012). The immune response to *Trypanosoma cruzi*: role of Toll-like receptors and perspectives for vaccine development. *Journal of Parasitology Research*.

[96] Valkov E., Stamp A., DiMaio F. (2011). Crystal structure of Toll-like receptor adaptor MAL/TIRAP reveals the molecular basis for signal transduction and disease protection. *Proceedings of the National Academy of Sciences of the United States of America*.

[97] Ferwerda B., Alonso S., Banahan K. (2009). Functional and genetic evidence that the Mal/TIRAP allele variant 180L has been selected by providing protection against septic shock. *Proceedings of the National Academy of Sciences of the United States of America*.

[98] Rudd K. E., Johnson S. C., Agesa K. M. (2020). Global, regional, and national sepsis incidence and mortality, 1990-2017: analysis for the Global Burden of Disease Study. *Lancet*.

[99] Cristofaro P., Opal S. M. (2003). The Toll-like receptors and their role in septic shock. *Expert Opinion on Therapeutic Targets*.

[100] Rajpoot S., Wary K. K., Ibbott R. (2021). TIRAP in the mechanism of inflammation. *Frontiers in Immunology*.

[101] Rajpoot S., Kumar A., Zhang K. Y. J., Gan S. H., Baig M. S. (2022). TIRAP-mediated activation of p38 MAPK in inflammatory signaling. *Scientific Reports*.

[102] Yong H.-Y., Koh M.-S., Moon A. (2009). The p38 MAPK inhibitors for the treatment of inflammatory diseases and cancer. *Expert Opinion on Investigational Drugs*.

[103] Rajpoot S., Srivastava G., Siddiqi M. I. (2022). Identification of novel inhibitors targeting TIRAP interactions with BTK and PKC*δ* in inflammation through an in silico approach. *SAR and QSAR in Environmental Research*.

[104] Hall N. B., Igo R. P., Malone L. L. (2015). Polymorphisms in *TICAM2* and *IL1B* are associated with TB. *Genes and Immunity*.

[105] Hawn T. R., Day T. A., Scriba T. J. (2014). Tuberculosis vaccines and prevention of infection. *Microbiology and Molecular Biology Reviews*.

[106] Faridgohar M., Nikoueinejad H. (2017). New findings of Toll-like receptors involved in *Mycobacterium tuberculosis* infection. *Pathogens and Global Health*.

[107] Matsumiya M., Stylianou E., Griffiths K. (2013). Roles for Treg expansion and HMGB1 signaling through the TLR1-2-6 axis in determining the magnitude of the antigen-specific immune response to MVA85A. *PLoS One*.

[108] Bulut Y., Michelsen K. S., Hayrapetian L. (2005). Mycobacterium tuberculosis heat shock proteins use diverse Toll-like receptor pathways to activate pro-inflammatory signals. *The Journal of Biological Chemistry*.

[109] Wang Y., Liu L. (2016). The membrane protein of severe acute respiratory syndrome coronavirus functions as a novel cytosolic pathogen-associated molecular pattern to promote beta interferon induction via a Toll-like-receptor-related TRAF3-independent mechanism. *mBio*.

[110] Totura A. L., Whitmore A., Agnihothram S. (2015). Toll-like receptor 3 signaling via TRIF contributes to a protective innate immune response to severe acute respiratory syndrome coronavirus infection. *mBio*.

[111] Gralinski L. E., Menachery V. D., Morgan A. P. (2017). Allelic variation in the Toll-like receptor adaptor protein Ticam2 contributes to SARS-coronavirus pathogenesis in mice. *G3 Genes|Genomes|Genetics*.

[112] Zhou R., To K. K.-W., Wong Y.-C. (2020). Acute SARS-CoV-2 infection impairs dendritic cell and T cell responses. *Immunity*.

[113] Bastard P., Rosen L. B., Zhang Q. (2020). Autoantibodies against type I IFNs in patients with life-threatening COVID-19. *Science*.

[114] Oliva A., Cammisotto V., Cangemi R. (2021). Low-grade endotoxemia and thrombosis in COVID-19. *Clinical and Translational Gastroenterology*.

[115] Li Y., Chen M., Cao H., Zhu Y., Zheng J., Zhou H. (2013). Extraordinary GU-rich single-strand RNA identified from SARS coronavirus contributes an excessive innate immune response. *Microbes and Infection*.

[116] van der Made C. I., Simons A., Schuurs-Hoeijmakers J. (2020). Presence of genetic variants among young men with severe COVID-19. *JAMA*.

[117] Greulich W., Wagner M., Gaidt M. M. (2019). TLR8 is a sensor of RNase T2 degradation products. *Cell*.

[118] Avcilar H., Eken A. (2020). Could imiquimod (Aldara 5% cream) or other TLR7 agonists be used in the treatment of COVID-19?. *Medical Hypotheses*.

[119] Angelopoulou A., Alexandris N., Konstantinou E. (2020). Imiquimod - a toll like receptor 7 agonist - is an ideal option for management of COVID 19. *Environmental Research*.

[120] Vlach J., Bender A. T., Przetak M. (2021). Discovery of M5049: a novel selective TLR7/8 inhibitor for treatment of autoimmunity. *The Journal of Pharmacology and Experimental Therapeutics*.

[121] EMD Serono Research & Development Institute, Inc (2020). ‘A phase II, randomized, double-blind, placebo-controlled study to evaluate the safety and efficacy of M5049 in hospitalized participants with COVID-19 pneumonia’, clinicaltrials.gov, Clinical trial registration study/NCT04448756. NCT04448756.

[122] Parija (2009). *Textbook of Microbiology & Immunology*.

[123] Chaby R. (2010). *Des Endotoxines Aux Lipopolysaccharides: Structures, Activités Cellulaires et Effets Physiopathologiques*.

[124] Angus D. C., Linde-Zwirble W. T., Lidicker J., Clermont G., Carcillo J., Pinsky M. R. (2001). Epidemiology of severe sepsis in the United States: analysis of incidence, outcome, and associated costs of care. *Critical Care Medicine*.

[125] Bone R. C. (1993). Gram-negative sepsis: a dilemma of modern medicine. *Clinical Microbiology Reviews*.

